# Identification and Characterization of Angiogenesis Targets through Proteomic Profiling of Endothelial Cells in Human Cancer Tissues

**DOI:** 10.1371/journal.pone.0078885

**Published:** 2013-11-13

**Authors:** Mehdi Mesri, Charlie Birse, Jenny Heidbrink, Kathy McKinnon, Erin Brand, Candy Lee Bermingham, Brian Feild, William FitzHugh, Tao He, Steve Ruben, Paul A. Moore

**Affiliations:** Celera, Alameda, California, United States of America; Center for Molecular Biotechnology, Italy

## Abstract

Genomic and proteomic analysis of normal and cancer tissues has yielded abundant molecular information for potential biomarker and therapeutic targets. Considering potential advantages in accessibility to pharmacological intervention, identification of targets resident on the vascular endothelium within tumors is particularly attractive. By employing mass spectrometry (MS) as a tool to identify proteins that are over-expressed in tumor-associated endothelium relative to normal cells, we aimed to discover targets that could be utilized in tumor angiogenesis cancer therapy. We developed proteomic methods that allowed us to focus our studies on the discovery of cell surface/secreted proteins, as they represent key antibody therapeutic and biomarker opportunities. First, we isolated endothelial cells (ECs) from human normal and kidney cancer tissues by FACS using CD146 as a marker. Additionally, dispersed human colon and lung cancer tissues and their corresponding normal tissues were cultured *ex-vivo* and their endothelial content were preferentially expanded, isolated and passaged. Cell surface proteins were then preferentially captured, digested with trypsin and subjected to MS-based proteomic analysis. Peptides were first quantified, and then the sequences of differentially expressed peptides were resolved by MS analysis. A total of 127 unique non-overlapped (157 total) tumor endothelial cell over-expressed proteins identified from directly isolated kidney-associated ECs and those identified from *ex-vivo* cultured lung and colon tissues including known EC markers such as CD146, CD31, and VWF. The expression analyses of a panel of the identified targets were confirmed by immunohistochemistry (IHC) including CD146, B7H3, Thy-1 and ATP1B3. To determine if the proteins identified mediate any functional role, we performed siRNA studies which led to previously unidentified functional dependency for B7H3 and ATP1B3.

## Introduction

Angiogenesis is the growth of new blood vessels from pre-existing ones and is an important natural process occurring in the body, both in health and in disease. Normal physiological angiogenesis occurs in adults during wound healing and endometrial regeneration during the menstrual cycle. However, pathological excessive angiogenesis can also occur in conditions such as in cancer, diabetic blindness, age-related macular degeneration and chronic inflammatory conditions [1]–[3].

It has long been known that the endothelium constituting blood vessels and surrounding stroma in tumors differ from that in normal tissues, but only recently these differences have begun to be characterized at the molecular level [4], [5]. Blocking abnormal blood vessels associated with cancer and other diseases using antiangiogenic agents has become a major therapeutic strategy. Because angiogenesis is required for normal physiological processes, markers that can distinguish physiological and pathological angiogenesis are needed in order to selectively deliver antiangiogenic agents to diseased tissues minimizing the potential side effects.

Target proteins located around tumor blood vessels and in the stroma are particularly suited for targeted anticancer strategies in view of their accessibility for intravenously administered therapeutics [4], [6]. Strategies for the identification of tumor-associated endothelial markers include *in vitro* ECs isolates exposed to culture conditions mimicking those in normal and tumor tissues [7], global profiling of gene transcripts [8], [9], bioinformatics analysis of expressed sequence tags [10], *in vivo* targeting using phage display peptide libraries [11], [12], silica coating procedure followed by stripping of membrane [13] and *in vivo* biotinylation methods [14].

A technical limitation in molecular profiling of ECs is that they represent a small percentage of the cells in the tissue. We have developed a methodology for the extraction, identification and large-scale mapping of the cell-surface proteome of microvascular endothelium as it exists *in vivo* in human kidney tumors and their adjacent normal tissues. This methodology is based on flow cytometric staining of vascular organs with known markers of ECs. Stained cells can be purified efficiently by cell sorting. Upon cell suface protein capture and tryptic digestion, the resulting proteolytic peptides are subjected to liquid chromatography – mass spectrometry (LC/MS) in order to identify the corresponding proteins. A comparative analysis of proteins identified in tissue specimens can reveal differences in the expression in different organs or conditions e.g. normal versus cancer. Moreover we analyzed *ex-vivo* cultured cells obtained from cancerous and adjacent normal human lung and colon microvascular ECs.

## Methods

### Chemicals and Materials

Chemical reagents were purchased from Aldrich-Sigma (St. Louis, MO). POROS R2 column (POROS R2/10, 4.6×50 mm) and POROS MC column (2.1×30 mm, IMAC column) were purchased from Applied Biosystems (Framingham, MA) and reversed-phase HPLC columns were obtained from Vydac (Hesparia, CA). DC protein assays were purchased from BioRad (Richmond, CA). Modified trypsin was purchased from Promega (Madison, WI). Antibodies against CD146, CD31, CD45, EpCAM, CD105, CD62E, Thy-1 (CD90) and B7H3 (CD276) were purchased from BD Biosciences. Dil-Ac-LDL was purchased from Biomedical Technologies Inc, MA.

### Tissue processing and phenotypic characterization

Human kidney, colon, lung and gastric tissues were obtained from commercial sources shortly after surgical removal. Commercial sources include Asterand (Detroit, MI, www.asterand.com) and BIOOPTIONS (Brea, CA, www.biooptions.com). Normal tissue was taken from regions >10 cm away from bulk tumor tissue that had clearly defined margins. The tissues were weighed, cut into 1 cm cubes with scissors, rinsed, and minced into 1 mm pieces. The tissue was then incubated with tissue digestion medium containing collagenase type I, hyaluronidase, DNase I, CaCl_2_, and dispase for one hour at 37°C with constant agitation. Any undigested tissue was filtered through a 40-µm mesh, and red cells removed by incubation in ACK lysis buffer for 5 min. followed by cell washes and cell count. Aliquots of single cell suspensions were stained with CD146, CD31, CD45, and EpCAM, to determine the profile of endothelial, epithelial and infiltrating leukocyte cells. The EpCAM negative, CD146 positive endothelial content of cell population were then sorted and isolated by a MoFlo cell sorter. For flow cytometry, ECs were stained with Thy-1 or B7H3 antibodies (BD Biosciences) or control, and analysis was performed on an LSR I (BD Biosciences) flow cytometer.

### *In vitro* culture of normal and tumor colon and lung ECs

Aliquots of single cell suspensions from commercially obtained (above) normal and tumor colon and lung tissues were prepared as described above. The endothelial cell population was then positively selected using Dynal's CD31 Endothelial Cell beads per product insert. The selected cells were seeded in T-flasks with Lonza's EGM™-2-MV media. This media was developed to enhance endothelial cell growth (Cambrex). The growth media was changed every 2–3 days until culture reached 90–100%. The cells were harvested using Lonza's ReagentPack™. The flasks were washed with Hepes buffered saline solution followed by trypsin/EDTA incubation to release the cells and a trypsin neutralizing solution. The positive selection of the endothelial cell was repeated to further purify the endothelial cell population. The selected cells were seeded in T-flasks with Lonza's EGM™-2-MV media. These primary cells did not lead to generation of cell lines and their limited expansion capacity provided sufficient cells only to complete experiments described here.

### Cell lysate and peptide preparation

Purified ECs were washed and incubated with 1mM sodium periodate at 4°C for 10 minutes to oxidize glycoproteins [15]. Cells were washed and checked for viability. Cells were greater than 85% viable as determined by PI exclusion using flow cytometry. Cells were lyzed in lysis buffer containing 0.05 M HEPES, pH 7.0, 150 mM NaCl, 2%SDS, 5 mM EDTA, 0.5% NP40 and protease inhibitor cocktail (Sigma) by vortexing and shearing through an 18-gauge needle. Protein concentration was then determined using DC protein assay reagent kit (BioRad, Hercules, CA). Oxidized glycoproteins were then conjugated to hydrazide resin (Bio-Rad) at 4°C overnight [16]. After washing sequentially with: 2 M NaCl, 2% SDS, 200 mM propanolamine (0.1 M NaOAc, pH 5.5), 40% ethanol and 80% ethanol; bound proteins were reduced in 2.5 mM dithiothreitol (DTT) (BioRad, Hercules, CA) for 1 h at 37°C and alkylated with ICAT (light only “C0”) according to the procedures recommended by the manufacturer (Applied Biosystems, Framingham, MA).

Alkylated proteins were digested overnight at 37°C using sequencing grade, modified trypsin (Promega, Madison, WI) with an enzyme to substrate ratio of 1∶25. Tryptic digests were desalted using 3-cm3 Oasis HLB solid-phase extraction columns (Waters, Milford, MA) and dried in vacuo.

### Cysteines and glycopeptides capture and sample cleanup

Peptides were reconstituted in a solution of 10% acetonitrile in PBS. Peptides were loaded onto an avidin column (2 mL, Applied Biosystems, Foster City, CA) using a Vision workstation system (Applied Biosystems) with PBS. The avidin column was washed with 50 mM ammonium bicarbonate (EMD, Gibbstown, NJ), 20% methanol, followed by a water wash. Non-cysteine-containing peptides were washed onto an R2/10 column (4.6×50 mm, Applied Biosystems) with 5% acetonitrile in 0.1% TFA and eluted to the fraction collector with a step gradient to 95% acetonitrile, 0.1% TFA. The cysteine-containing peptides were eluted using 50% acetonitrile, 0.1% TFA (Pierce, Rockford, IL), collected, and vacuum-dried. Captured ICAT-labeled peptides were cleaved as described in the protocol provided by Applied Biosystems. Excess and cleaved ICAT reagent was removed using the same avidin/R2 cleanup described above. The R2/10 fractions (cysteine-containing peptides) were dried in vacuo and stored at −80°C before LC-MS analysis.

In addition to the cysteine-containing peptide fraction, peptides bound to the resin were also collected and analyzed. Release of peptides was achieved through PNGase-F digestion (New England BioLabs, Ipswich, MA). While we found some overlap between the proteins identified in the two fractions, analysis of both the cysteine-containing fraction and the resin-bound fraction resulted in complementary coverage of the cell surface protein population.

### Reversed phase LC/MS and LC/MS/MS

Reverse phase HPLC separation was performed on an Agilent 1100 system (Palo Alto, CA) set to a flow rate of 2 µL/min. The system was equipped with a C18 precolumn for desalting (50 mm ×0.15 mm), a switching valve, and a C18 analytical column (150 mm ×0.15 mm) for peptide separation. MS and MS/MS analyses were performed on a Q-Star mass spectrometer (MDS/Sciex, Toronto, Canada). The data acquisition time was set to 3 s per spectrum over a *m/z* range of 400 to 1500 Da for LC/MS analyses. The ion spray voltage was set at 5.2 kV.

### Peptide identification and quantitation

Peptide ion peaks from the LC/MS maps were detected with RESPECT^TM^ software (PPL, UK), and MASCOT (www.matrixscience.com) was used to search MS/MS spectra for peptide/protein identification with mass spectrometry protein sequence database (MSDB, Imperial College London, London, UK). Peptide ion peaks of LC/MS maps from normal and tumor samples were aligned based on mass-to-charge ratio (*m/z*), corrected retention time (RT) and charge state (*z*). The intensities of ions in each map were compared to the mean intensities of those ions across all maps in the alignment. A normalization factor was generated (using ions with mean intensities in the 10^th^–90^th^ percentiles) for each map, using unconstrained nonlinear optimization, minimizing the sum of the differences between intensities of each ion and the mean intensity for that ion across all maps. After intensity normalization, peptide ions with a differential expression of greater than 3-fold between tumor and normal samples were manually verified before LC-MS/MS-based peptide sequencing.

### Immunohistochemistry (IHC)

Immunohistochemistry was performed by LifeSpan Biosciences on formalin-fixed paraffin-embedded tissues using tissue microarrays. The cancer array consisted of homogeneous cancer cells. The tissues were deparaffinized, and antigen retrieval was done using EZ-retriever (Biogenex, San Ramon, CA). Samples were pre-blocked with non-serum protein block (DAKO A/S, Carpinteria, CA) for 15 minutes. Corresponding primary antibodies were incubated overnight at room temperature. Envision Plus system HRP (DAKO) was used for detection with 3, 3′-diaminobenzidine as substrate for horseradish peroxidase-conjugated secondary antibodies. Slides were then manually scored by one pathologist and were based on (i) staining intensity and (ii) staining frequency modifiers. The negative staining intensity was assigned the score of “0”, blush staining a score of “1”, faint staining a score of “2”, moderate staining a score of “3”, and strong staining a score of “4”. In addition, when 0.1% to 30% cells showed positive staining, the staining was quantified as rare or occasional, 30% to 75% of cells showing positive staining were quantified as “many or frequent”, and greater than 75% of cells staining positive were designated “most”. Hematoxylin was used as a counter-stain. Representative images were acquired using 40 X objective (400 X magnification).

### Immunofluorescence

Human umbilical vein endothelial cells (HUVECs, Cambrex) were grown on Lab-Tek chamber tissue culture slides. Cells were incubated with 10 µg/ml Dil-Ac-LDL at 37°C in normal media for 4 h. The media was then removed and the cells were washed once with probe-free media for 10 min, rinsed with PBS, and then fixed with 10% buffered formalin phosphate for 5 min. Coverslips were mounted over the slide with a drop of 10% PBS in glycerol prior to viewing.

### RNA Interference

Individual duplex synthetic siRNA specific for B7H3 and ATP1B3, were purchased from Dharmacon (Lafayette, CO). For siRNA transfection, HUVECs or human microvascular endothelial cells (HMVECs, Cambrex) were seeded into 96-well tissue culture plates at a density of 10,000 cells/well. Transfections were performed using Lipofectamine 2000 and Plus reagent (Invitrogen) according to the manufacturer's protocol. Knockdown of B7H3 mRNA levels was monitored by quantitative RT-PCR 1 day after transfection using TaqMan Gene Expression Assay (Applied Biosystems). Percent mRNA expression for knockdown validation was calculated using the ΔΔCt method [17]. RPLP0 served as the endogenous control to normalize for template load, and cells transfected with negative control siRNA were the reference sample. Cell growth was assessed 3 days after transfection using alamar blue reagent (Invitrogen) and SPA-thymidine incorporation kit from GE Healthcare (Piscataway, NJ). Cell apoptosis was measured by caspase 3/7 activity kit from Promega (Madison, WI).

## Results

### Evaluation of endothelial content in multiple cancer types

**Figure 1:**
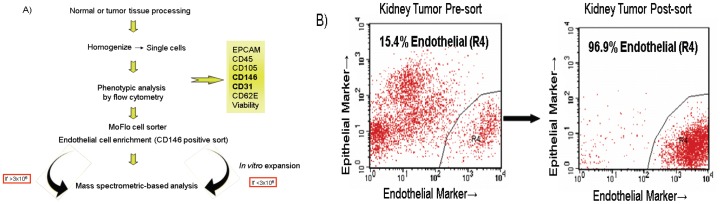
A) Schematic representation of ECs isolation from human kidney tissues. B) For isolation of pure EC populations from collagenase dispersed tissues, the EpCAM negative, CD146 positive endothelial content of cell population were sorted, isolated and enriched by a MoFlo cell sorter. Cells were submitted for LC-MS and feature detection either directly or indirectly after expansion in culture.

**Figure 2:**
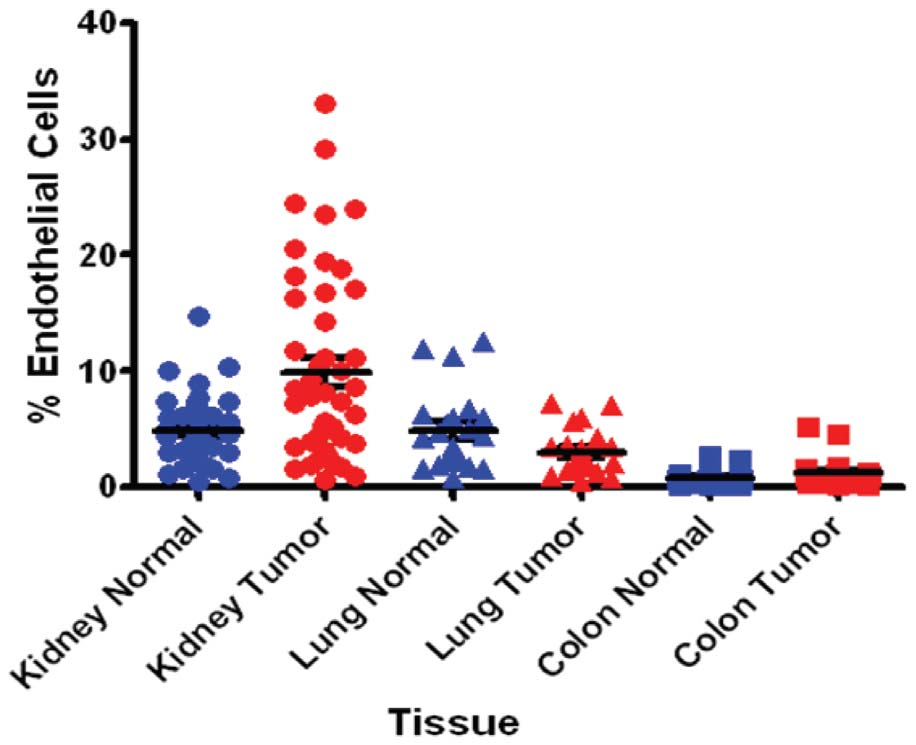
Endothelial Cell Content of Tissues. Single cells following tissue processing from tumor and normal adjacent from kidney, lung, and colon tissues were stained with anti-CD146 Ab and analyzed for EC presence.

To evaluate the relative EC content of tumors across multiple cancer type indications, single cells obtained following tissue processing from tumor and normal adjacent tissues from kidney, lung, colon and gastric cancers and were analyzed for EC presence (Fig 1). ECs are enmeshed in a complex tissue containing an extracellular matrix of variable composition and multiple non-endothelail cell types such as epithelial cells and leukocytes. Our initial attempts to purify ECs involved antibody recognition of conventional known EC cell surface markers including CD31, CD105, and CD146. However, CD31 proved to be suboptimal because of its cross-reactivity with hematopoietic cells. CD105 also can be expressed by activated monocytes and also proved suboptimal in detecting all sources of ECs. Anti-CD146 showed maximal EC recognition as confirmed by dual staining of CD31 and CD146 by FACS and positive acetylated LDL (Dil-Ac-LDL) uptake by immunocytochemistry in cultured ECs derived from colon and lung tissues. Anti-CD146 Ab was therefore used for EC sorting, isolation, enrichment and relative estimation of ECs content of tumor tissues. Our analyses indicated that kidney tissues contained the highest percentage (0.6–33.1) of ECs (Fig 2) followed by lung (0.8–7.2), colon (0.2–5.1) and gastric tumors (0.2–1, not shown in Fig 2). A total of 46 matched tumor and adjacent normal kidney tissues were analyzed representative of all four stages of kidney cancer. The content of normal kidney ECs varied from 1.1–14.7% compared to 0.6–33.1% in kidney cancers. The elevated content of ECs in kidney tumors was also evident when compared to each corresponding matched normal adjacent ECs suggesting that kidney tumor ECs may be more proliferative compared to their more quiescent normal adjacent ECs. This trend however was not evident in lung and colon tissues. Since protein quantity is a limiting factor for MS analysis, we therefore performed our discovery effort on kidney cancer which provided the highest ECs recovery from tissues.

### Sample preparation and MS quality control

**Figure 3:**
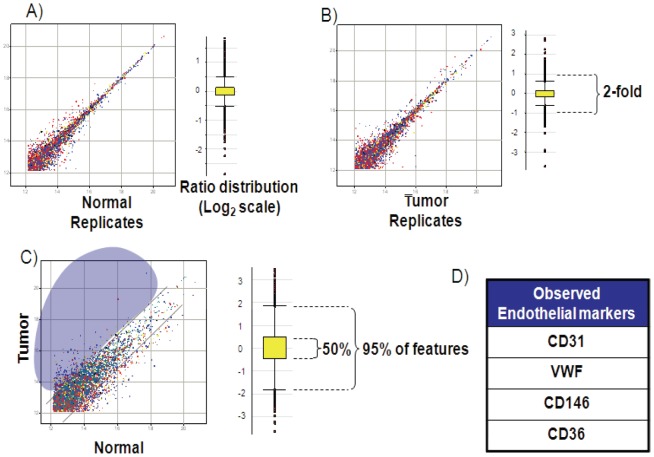
Mass Spectrometry (MS) analysis and quality control. Scatter plots of common features from normal and tumor samples and differential analysis using decoupled maps are shown. Relative expression levels are determined by aligning peptide ion features from different MS experiments (LC/MS maps). The log2 intensities of common features detected in (**A**) process replicates of the control sample; (**B**) process replicates of the tumor sample; (**C**) control vs. tumor samples are plotted. The corresponding box plot (in log2 scale) of ratio distribution for each dataset is shown next to the scatter plot. The box contains 50% of the features, where 95% of the features are within the horizontal bars. (**D**) Prevalence of well known EC surface proteins was investigated and representatives are shown.

The project focused discovery on markers containing short, tryptically-cleaved 5–25 amino acid peptides encoding either a cysteine residue or an N-linked glycosylation site, providing broad coverage of the proteome. To evaluate the MS system suitability, an assessment of reproducibility was performed for determining the minimum reliable ratio of peptide/protein levels for further quantitative analysis. Cysteine-containing peptides from the tryptic digest were enriched by affinity chromatography, and LC-MS maps of the peptide mixtures from two samples were generated. In our experiments the ICAT (light only) label served as a handle to pull out cysteine containing peptides, thus simplifying the peptide mixture. While the samples analyzed technically contain a label, our analysis is essentially what is known as a ‘label-free’ method in that peptide ion peak areas from a particular sample and LCMS map are compared to those of a different sample and LCMS map for relative quantification. Thus we term our experiments “decoupled,” meaning that the labeled samples are not combined into one mass spectrometric analysis as is typically done in an ICAT or labeling workflow. The feature lists of, for example, Normal replicate 1 and Normal replicate 2 were aligned to generate an intensity matrix. Fig. 3 shows the scatter plot of log2-transformed intensities (log2 Int) of the common features in the decoupled experiments (replicate 1 vs replicate 2). Log2 ratios (ratio  =  ion intensity in sample 1/ion intensity in sample 2) of all common features were calculated. The summary statistics are shown with the boxplot diagram. For the ratio distribution of the decoupled replicates, 2SDUL (upper limit of 2 standard deviations (SD) from the mean), 2SDLL (lower limit of 2SD from the mean), and mean values in linear scale are shown in Fig. 3. This reproducibility assessment indicated that for replicate experiments (Normal or Tumor) 95% of the total common features have intensities within a ∼2-fold difference. Therefore, any difference in expression ratio that is >2-fold may be considered as differentially expressed with 95% confidence. In this study however, ratios that were >3-fold were considered as differentially expressed.

### Heat map of global peptide analysis in human kidney tumor endothelium

A total of 6 tumors and 2 normal adjacent kidney samples were processed for tumor relative over-expression analysis. A total of 74 cell surface/secreted proteins were identified by MS with minimum 3 fold higher intensity in kidney tumor-associated endothelium compared to normal endothelium.

**Figure 4:**
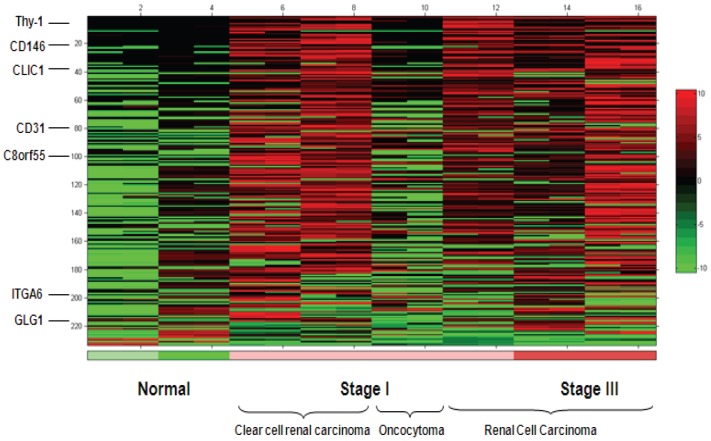
Global peptide analysis identified over-expressed proteins in kidney tumor tissue endothelium. The heat map presents the analysis of peptide intensities for the 233 peptides identified in kidney tumor endothelium and sorted so that the most differentially-expressed peptides are at the top. The display colors were determined for each row separately by assigning black to the median intensity in the row, green to the lowest intensity in the row, and red to the highest intensity.

The heatmap rows were sorted by the ratio of the mean intensity in the tumor samples to the mean intensity of the normal samples. The heat map presents the analysis of 233 cysteine containing peptide intensities identified in kidney tumor endothelium and sorted so that the most differentially-expressed peptides are at the top (Fig 4). Rows were only included if there was at least one MS/MS identification of an ion in the row. The display colors were determined for each row separately by assigning black to the median intensity in the row, green to the lowest intensity in the row, and red to the highest intensity. In rows with a median of zero, the zero values are displayed as black. Examples of identified proteins are depicted on the heat map.

### MS analysis of proteins with elevated expression in *in vitro* cultured human colon and lung tumor endothelia

**Figure 5:**
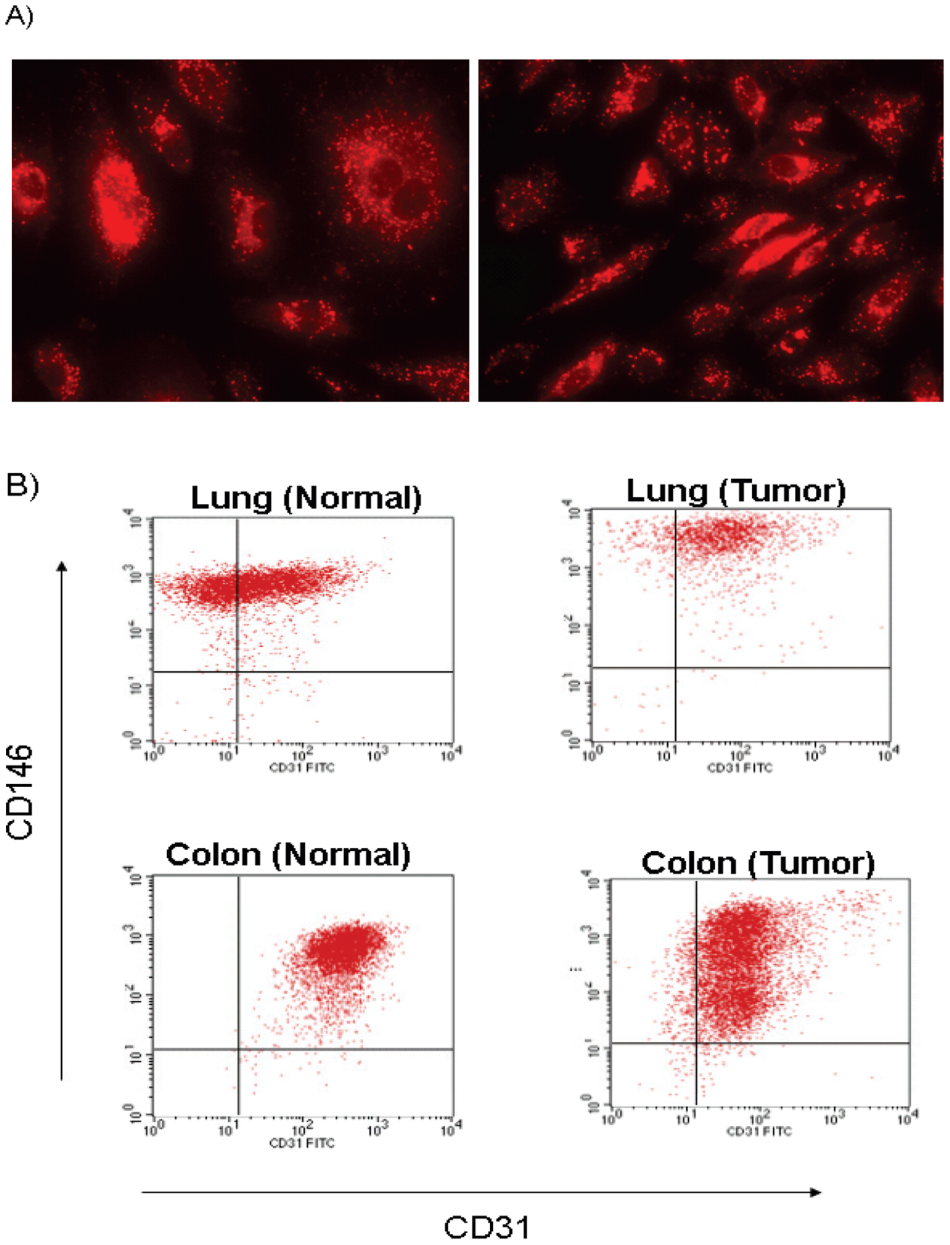
Phenotypic characterization of *Ex Vivo* cultured endothelial cells. (**A**) Intense punctate fluorescence demonstrating the uptake of DiI-Ac-LDL in normal lung microvascular ECs. (**B**) Cultured normal and tumor tissue-derived ECs from colon and lung were stained with EC specific markers CD146 and CD31, and subjected to flow cytometry.

We further cultured and expanded normal and tumor ECs *in vitro* from cancer type indications that did not yield adequate endothelial proteins from direct *in vivo* isolation for MS analysis. These indications included colon and lung cancers. Identity of these cells as ECs was confirmed by dual staining of CD31 and CD146 by FACS and positive Dil-Ac-LDL uptake by immunocytochemistry (Fig 5). These cells were negative for alpha actin expression, a typical marker of smooth muscle cells. Interestingly, only a fraction (20–30%) of *in vitro* cultured ECs derived from lung and colon tumors stained for Dil-AC-LDL uptake by immunocytochemistry (not shown) despite their positive expression of CD31 and CD146 as observed by FACS (Fig 5b). In contrast, all of the *in vitro* cultured ECs derived from normal lung and colon stained for Dil-AC-LDL uptake by immunocytochemistry (Fig 5a) and stained positive for CD31 and CD146 as observed by FACS (Fig 5b). This may suggest that tumor ECs may differ phenotypically from their normal counterparts. A total of 56 colon and 27 lung cell surface/secreted proteins were identified with >3 fold over-expression by MS in tumor-associated endothelium compared to normal.

A summary of ECs target discovery across the three cancer type indications is shown in Table 1. A total of 127 unique non-overlapped (157 total) tumor endothelial cell over-expressed proteins identified from directly isolated kidney-associated ECs and those identified from *ex-vivo* cultured lung and colon tissues. Our analysis indicated that of proteins identified, 4 proteins overlapped across all three cancer types; 17 proteins overlapped between kidney and colon; 9 proteins overlapped between kidney and lung; and 8 proteins overlapped between lung and colon cancers. The list of MS identified targets, their tissue source of detection and expression ratio is depicted in Table S1. In analyzing over-expressed proteins, we identified known ECs markers including VWF (5-fold over normal, maximum ratio), PECAM (CD31, 36-fold), CD36 (16-fold), and MCAM (CD146, 15 fold).

**Table 1:** Summary of endothelial cell target discovery.

Identification	EC Tumor Type	# of Targets
ECs derived from tumor tissue followed by MS analysis	Kidney	74
ECs derived from tumor tissue and cultured/propagated *ex vivo *followed by MS analysis	Colon	56
	Lung	27
	Totals	157

Number of tumor over-expressed proteins identified by MS from directly isolated kidney-associated endothelial cells and those identified from *ex-vivo* cultured lung and colon tissues are shown.

### Target Validation

**Figure 6:**
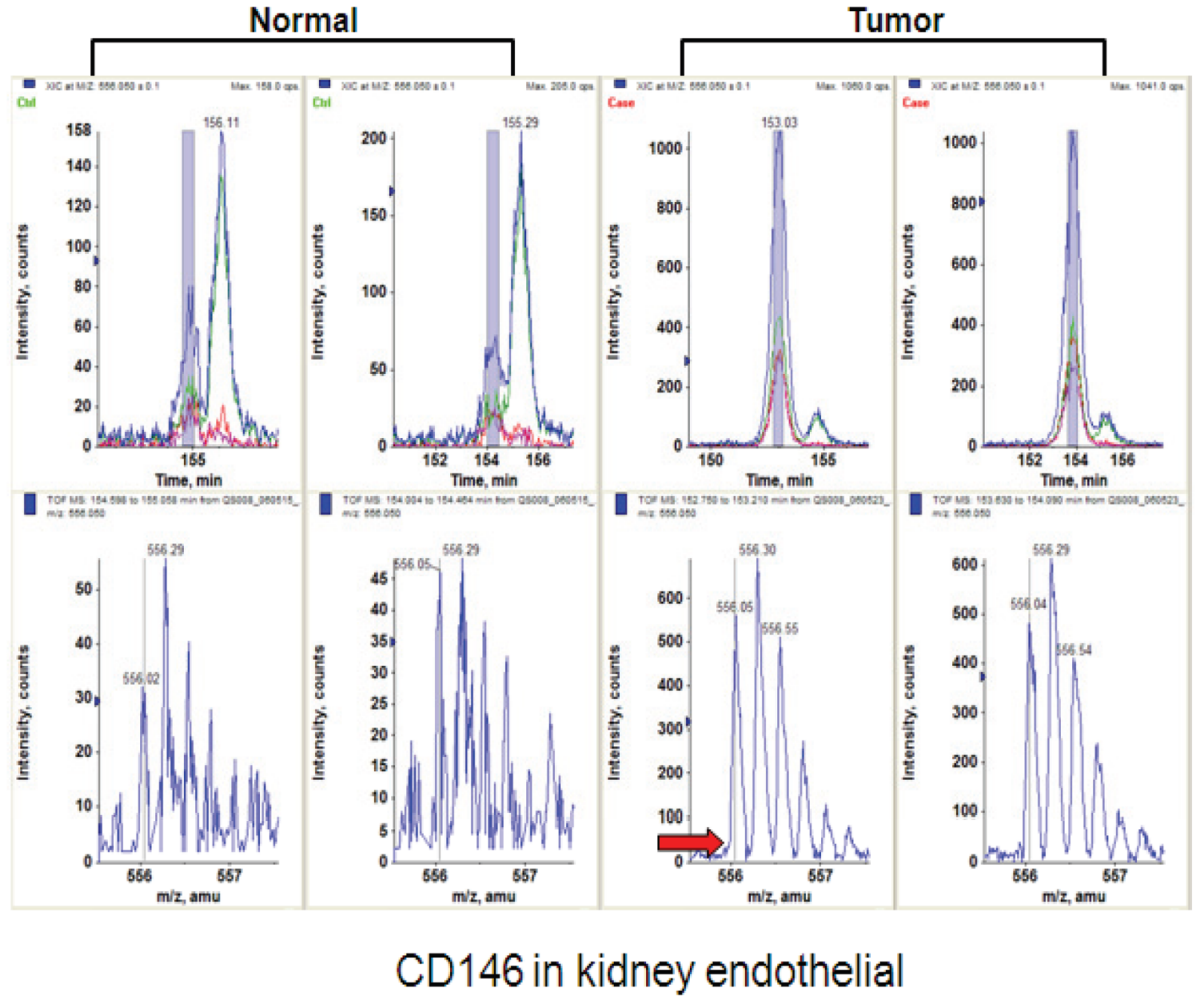
MS analysis of CD146 in tumor ECs. The top panel shows the extracted ion chromatograms of kidney ECs samples from normal and tumor samples. The mass spectra are shown in the lower panel. The red arrow shows the intensity level in normal tissue sample.

To validate proteins identified by MS, we performed additional confirmatory studies including immunohistochemistry (IHC), flow cytometry and RNAi-mediated effect on EC proliferation and/or apoptosis on a subset of proteins that were selected based on criteria such as druggability of the proteins by antibodies, novelty, and intensity of tumor over-expression. Representative data from a number of proteins are presented below. A representative MS analysis of peptide ions from CD146 is shown in Fig. 6. Our MS analysis identified 11 peptides derived from CD146 across 7 different disease indications including 4 peptides in kidney ECs (data not shown).

**Figure 7:**
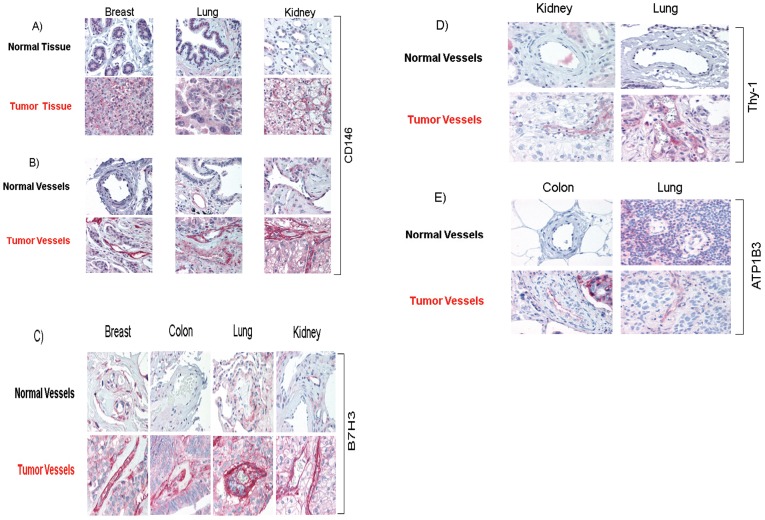
Overexpression of CD146, B7H3, Thy-1 and ATP1B3 proteins in tumor cells and/or endothelial cells is confirmed by IHC. (**A**) IHC images of normal and carcinoma samples. Over-expression of CD146 is found in tumor cells while normal epithelium was uniformly negative in multiple oncology indications. (**B**) Images indicate significant over-expression of CD146 in ECs of carcinoma vessels compared to normal samples. (**C**) Significant over-expression of B7H3 in ECs of carcinoma vessels compared to normal samples is depicted. (**D**) Significant over-expression of Thy-1 in carcinoma vessels compared to normal is shown. (**E)** IHC images of ECs indicate significant over-expression of ATP1B3 in carcinoma vessels compared to normal samples.

Expression intensity of a number of proteins including CD146, B7H3, Thy-1 and Sodium/potassium-transporting ATPase subunit beta-3 (ATP1B3) were evaluated by IHC. We analyzed paraffin sections taken from multiple independent patient tissues from a variety of cancer types including breast, lung, colon, kidney, or normal adjacent tissues. All samples represented unrelated cases than those used for MS analyses. Representative IHC stainings are depicted in Fig 7. IHC was performed on sections from multiple normal and carcinoma samples including breast, lung and kidney specimens. Interestingly, over-expression of CD146 is found in tumor cells while normal epithelium was negative in multiple cancer types (Fig. 7a). It is noteworthy that our sorting protocol excluded CD146 and EpCAM positive epithelial cells from proteomic analysis. CD146 over-expression was frequently detected in the endothelium of tumor cells while normal epithelium was uniformly negative (Fig. 7b). IHC images of ECs indicate significant over-expression of B7H3 in breast, colon, lung and kidney carcinoma blood vessels compared to normal vessels (Fig. 7c). Furthermore, in many of the tumors examined, we detected over-expression of B7H3 protein by the tumor cells themselves (data not shown). For Thy-1, IHC images also indicated significant over-expression in kidney, and lung carcinoma vessels compared to normal vessels (Fig. 7d). IHC images indicated over-expression of ATP1B3 in carcinoma vessels compared to normal samples (Fig. 7e). Over-expression of ATP1B3 was also demonstrated in tumor cells compared to normal samples in colon and lung cancers (data not shown).

**Figure 8:**
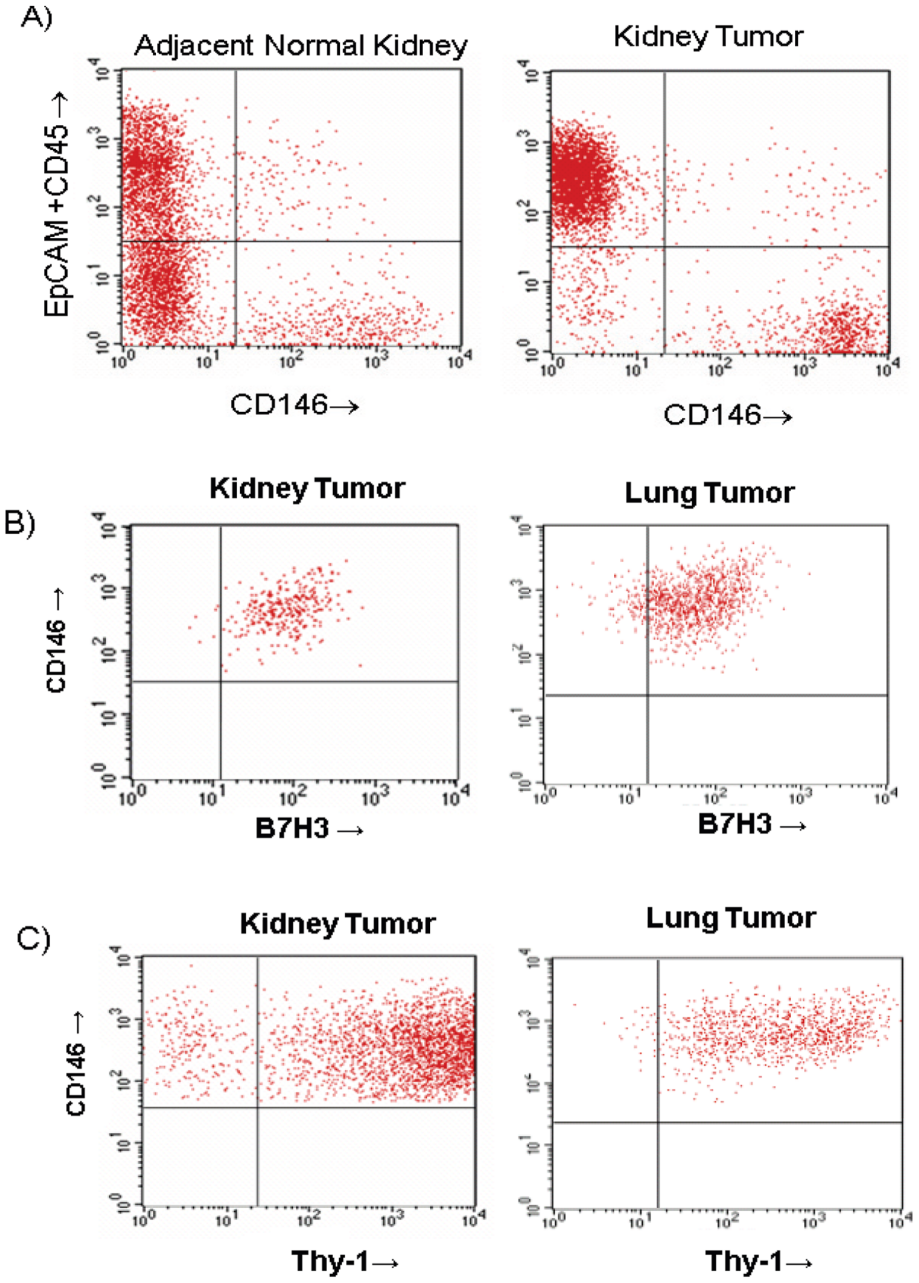
Phenotypic characterization of cellular content of tumors. (**A**) Over-expression of CD146 in kidney tumor tissue cells was confirmed by flow cytometry. (**B**) and (**C**) respectively confirm B7H3 and Thy-1 over-expression in kidney and lung tumor tissue endothelia by flow cytometry.

**Figure 9:**
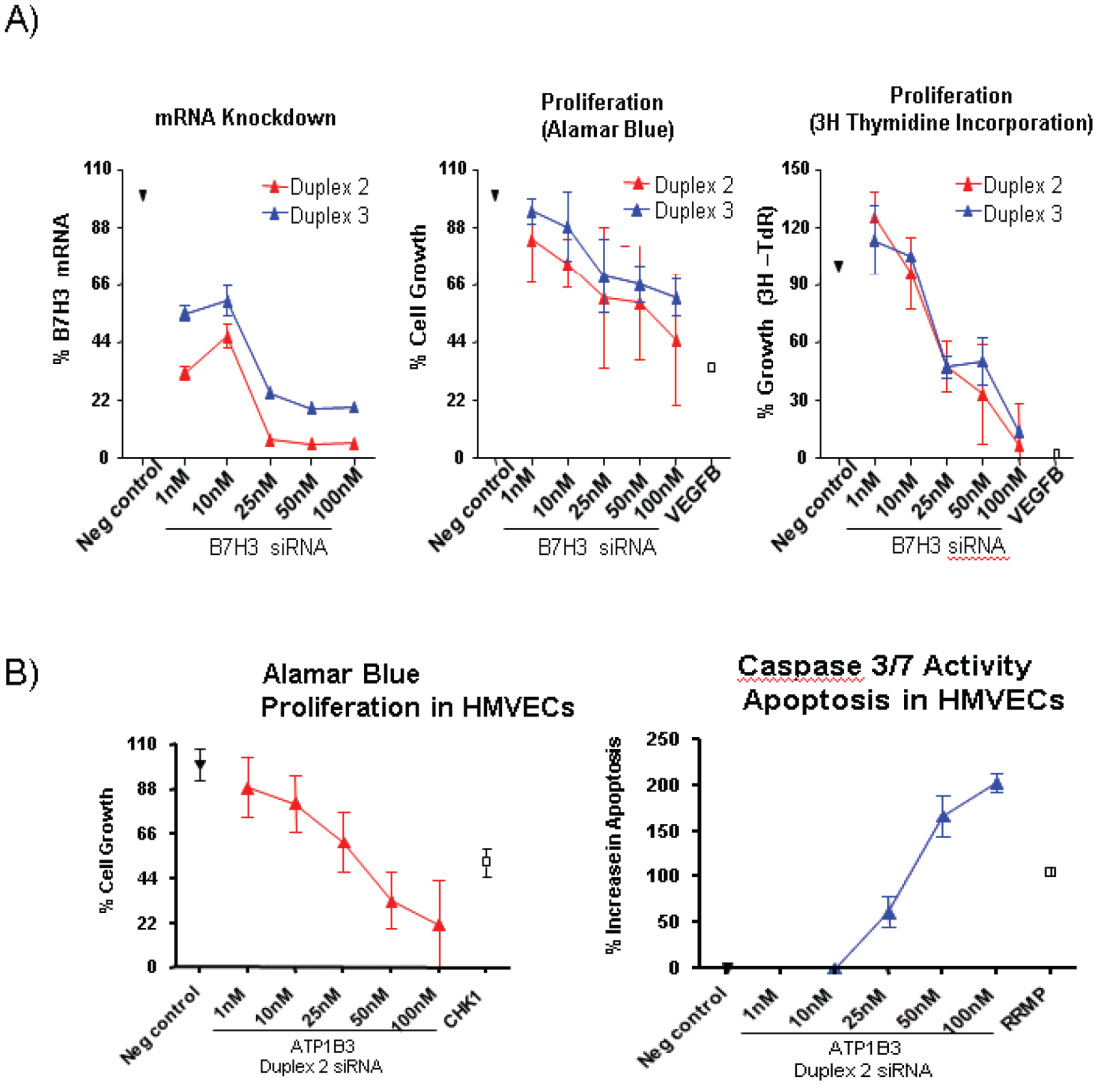
Functional activity of selected targets. (**A**) mRNA knockdown of B7H3 inhibits proliferation in HUVECs in a titration dependent manner. A representative screen and a titration experiment with duplexes 2 and 3 in HUVECs are shown. Proliferation was monitored by using the Alamar Blue assay, and SPA [3H]. Thymidine uptake assay system. (**B**) mRNA knockdown of ATP1B3 inhibits proliferation and increases apoptosis in HMVECs in a titration dependent manner. A titration experiment with duplexes 2 in HMVECs is shown. Proliferation was monitored by using the Alamar Blue assay, and apoptosis was measured by caspase 3/7 activity.

Additional confirmatory studies were conducted by flow cytometry. Fig. 8a represents FACS data on characterization of sorted cells within tumor indicating presence of epithelial cells and haematolymphoid cells with no expression of CD146 in kidney samples while there is significant over-expression of CD146 on endothelial component of tumor compared to adjacent normal kidney. Figs. 8b and 8c respectively indicate that CD146 positive endothelial cells of kidney and lung tumors express B7H3 and Thy-1 proteins. To evaluate potential functional role of above proteins, we performed siRNA analyses in cultured ECs. Because there were multiple limitations for conducting siRNA experiments in tumor ECs we used HUVECs or HMVECs as available model systems. The ability to maintain tumor derived ECs *ex-vivo* was limited in passage frequency and quantity. The ECs we were able to harvest was sufficient for only the MS analyses and not sufficient to maintain in culture long enough to support siRNA studies including the necessary optimization work needed to transfect with siRNA as well as having cells for the actual assays. Therefore we used proliferating HUVEC and HMVEC cultures that mimic more angiogenic neovasculature characteristics than established vasculature and are well established *in vitro* cell models for tumor neovasculature expressed targets such as B7H3 and ATP1B3. These analyses led to identification of functional activities in both HUVECS (for B7H3) and HMVECs (for ATP1B3) as described below. To determine if B7H3 plays a functional role in cell survival, HUVECs were treated with two independent siRNA duplexes targeting B7H3 in a titration dependent manner. Negative controls, included scrambled negative control siRNA, and transfection reagent-alone. Twenty-four hours following B7H3 siRNA transfection, more than 80% knockdown was observed particularly at 25 nM concentrations and above (Fig. 9a). Three days following siRNA transfection, a dose-dependent inhibition of cell growth, as measured by cellular metabolic activity (Alamar Blue), was observed for B7H3 with the phenotypic effects observed using independent siRNAs targeting different portions of each candidate mRNA (Fig. 9a). Similar inhibitory effects were observed for positive control VEGF-B. As anticipated, no inhibitory effects were observed for the negative control siRNA. Additional studies addressed whether inhibition of cell growth was due to a decreased number of cells undergoing DNA synthesis. As shown in Fig. 9a, siRNA targeting of B7H3 inhibits thymidine incorporation. The observed inhibition of DNA synthesis was evident for both independent duplexes as compared with the scrambled negative control. mRNA knockdown of ATP1B3 also inhibited proliferation and increased apoptosis in HMVECs in a titration dependent manner (Fig. 9b).

## Discussion

For many years, physiological and pathological angiogenesis have been known to be morphologically distinct [18]. However, the extent of differential expression between these cellular states has remained elusive. Most of the well-studied molecules that are thought to regulate tumor angiogenesis such as VEGF, bFGF, the angiopoietins, and their receptors also regulate normal physiological angiogenesis. To identify over-expressed tumor-associated endothelial proteins, we compared proteome profiles in endothelium derived from normal and tumor tissues. Global analysis of protein expression in tumor and normal endothelium is difficult because endothelium is embodied in a complex tissue consisting of vessel wall components, stromal cells, and epithelial cells. Moreover, only a small fraction of the cells within these tissues are endothelial [8]. To overcome these issues, we attempted to purify ECs from dispersed human tissues using CD146, an endothelial marker commonly used for this purpose [19]. This resulted in a considerable enrichment of ECs without contamination of the preparations by hematopoietic cells that may otherwise occur if had used CD31 [8]. The ECs purified using this protocol were essentially free of epithelial cells and hematopietic cells as judged by flow cytometry and further downstream from our MS analyses. While an important concept in tumor angiogenesis is that tumor endothelial cells are genetically normal and homogeneous, the endothelial cells in our study indicated a heterogeneous population particularly when compared between normal and tumor endothelial compartment. This may well be attributable to proteogenomical differences and antigenic/phenotypic heterogeneity between normal and tumor endothelial cells [20]. In addition to the microenvironment, epigenetic factors and tumor endothelial genetic instability may also play a role in mediating tumor endothelial heterogeneity [21]. The heterogeneous nature of endothelial cells were also evident from fractional (20–30% of cells) Dil-AC-LDL uptake by *in vitro* cultured ECs derived from lung and colon tumors versus entire cell staining of the *in vitro* cultured ECs derived from normal lung and colon specimens in our study (Fig. 5a). Such heterogeneity may provide important clues into vascular bed-specific therapies in cancer.

Human kidney cancer was chosen for direct isolation studies because our phenotypic analysis exhibited that this tumor indication has a relatively high frequency of endothelial content. Using direct isolation and capture of ECs from kidney tissues or their *in vitro* expansion from organs such as colon and lung tissues where limited ECs obtained, we were able to identify a considerable number of endothelial markers. Accessible proteins lining vascular structures in the normal tissues and in the solid tumor mass were purified by flow cytometry and identified using LC-MS and MS-MS methodologies, revealing 127 cell surface/secreted tumor over-expressed proteins. Of 127 total proteins, only 4 overlapped among the three endothelial-derived cancer types suggesting that ECs from different anatomical sites may over-express unique proteins and that they may be distinguished based on their unique protein expression signatures. Such organ-specific endothelial markers may hold a critical key to the selective delivery of therapeutic medicines to targeted anatomical sites. The anatomical distinctive over-expression may also confirm the notion that although all proteins expressed in tumor endothelium are expected to have some normal physiological function, they may not be expressed in all types of angiogenesis, or be expressed during certain developmental stages but are turned off in the adult. We do not however rule out the possibility that lung and colon ECs grown in culture may introduce proteomic alterations compared to those isolated directly from kidney tumor which may contribute to anatomical proteome differences.

Interestingly, we observed upregulation of CD146 in tumor ECs which is a highly glycosylated junctional adhesion molecule, involved in the control of vessel integrity. Recent results identify galectin-1 as a novel ligand for CD146 and this interaction protects endothelial cells against apoptosis induced by galectin-1 *in vitro* [22]. Beside its role in endothelial cell permeability and angiogenesis, CD146 over-expression has also been associated with survival signals such as Akt phosphorylation and down-modulation of Bad expression [23]. It is therefore possible to speculate that upregulation of CD146 in tumor ECs is a survival mechanism although additional studies are needed to substantiate this.

As an effort toward evaluating the therapeutic potential of some of endothelial targets in humans that we identified, we focused on B7H3, Thy-1 and ATP1B3, which were among the more differentially expressed cell surface tumor-specific endothelial markers identified by MS in this study. B7H3 is a recently identified member of the B7 family of immunoregulatory molecules that can be induced on T cells, B cells, and dendritic cells by a variety of inflammatory cytokines [24] and identified as the most differentially expressed cell surface tumor-specific endothelial marker by SAGE [9]. In addition to strong detection of B7H3 in kidney tumor endothelial cells, we also detected strong staining of the tumor vasculature in colon, lung and breast cancers consistent with a prior report [9]. Furthermore, in many of the tumors examined, we found B7H3 protein overexpression by the tumor cells themselves. The strong differential reactivity to multiple solid tumor tissues compared with normal tissues and lack of positive staining in human normal pancreas, lung, liver, kidney, and heart has also been reported recently by Loo *et. al.* [25]. Furthermore, vascular endothelium staining in renal cell carcinoma was also demonstrated [25]. In such cases, agents which target B7H3 may target both the tumor and stromal compartments simultaneously, thus resulting in an enhanced therapeutic efficacy. Interestingly, an anti–B7H3 mAb that mediates potent antitumor activity *in vitro* as well as in tumor xenograft studies has recently been developed. This mAb known as MGA271 has shown favorable safety profile in cynomolgus monkey toxicology studies and a phase I/IIa clinical study in patients with B7H3–positive metastatic or recurrent adenocarcinoma has been initiated [25]. Additionally using immunohistochemical tissue microarray analysis on tumor specimens, B7H3 has been shown to be expressed in the endothelium of tumor associated vasculature in ovarian carcinoma patients, and was associated with poor clinical outcome. B7H3 expression in tumor vasculature may be a reflection of tumor aggressiveness and has diagnostic and immunotherapeutic implications in multiple cancers [26].

Thy-1, a highly glycosylated GPI-anchored cell surface protein with a molecular mass ∼35 kDa, is a receptor on EC, belonging to the Ig superfamily, and is involved in adhesion of leukocytes to the endothelium [27]. In humans, Thy- 1 expression has been demonstrated in melanoma cells [28] and as a marker of cancer stem cells in glioma [29]. Furthermore Thy-1 has been shown on activated EC [30], fibroblasts, neuronal cells, a subset of peripheral CD34+ stem cells [27], lymphatic vessels [31] and as a marker of angiogenesis [32]. Furthermore, there is evidence that high metastatic tumor ECs express higher Thy-1 mRNA levels compared with low metastatic tumor ECs suggesting heterogeneity of ECs in tumors [20]. To our knowledge, the present study is first to establish that Thy-1 is overexpressed on blood vessels of multiple tumor types and suggests that Thy-1 may serve as a target for therapeutic intervention.

ATP1B3 (CD298) belongs to the subfamily of Na +/K+ -ATPases. Na +/K+ -ATPase is an integral membrane protein responsible for establishing and maintaining the electrochemical gradients of Na and K ions across the plasma membrane. This enzyme is composed of two subunits, a large catalytic subunit (alpha) and a smaller glycoprotein subunit (beta). The beta subunit regulates, through assembly of alpha/beta heterodimers, the number of sodium pumps transported to the plasma membrane. Recent studies indicates that the α- and β-subunits might independently be involved in cellular functions other than ion pumping [33]. For example, the β-subunit has been suggested to play a role in cell-cell adhesion. The amounts of the cell surface β-subunits increase when the cell density becomes higher, whereas the amount of the α-subunit does not change significantly. These results indicate that the quantity of the β-subunit in the plasma membrane is regulated depending on the cell density, and suggest the general involvement of the β-subunits in cell-cell adhesion. This notion is supported by the probability that the extracellular domain of the β-subunit may possess the Ig-fold structure for cell-cell adhesion [33]. Further functional data is needed to elucidate the significance of ATP1B3 over-expression on tumor ECs and its relevance as a potential angiogenesis target.

Supplemental validation data including generation of monoclonal antibodies, immunofluorescence analysis, and biodistribution studies in appropriate animal tumor models are required to demonstrate the potential of these putative vascular-associated antigens identified in this study. Many of the proteins identified in our study were also over-expressed in the same tumor indication epithelial cells which suggest these proteins have the potential to be used as doubled-barreled therapeutic targets. The validation data presented here with CD146 as a known endothelial marker indicates that our large scale proteomic mapping capabilities can provide a platform for identification of novel therapeutics.

While we developed proteomic methods that allowed us to focus our studies on the discovery of cell surface/secreted proteins, as they represent key antibody therapeutic and biomarker opportunities, the proteomic method could be modified for intracellular studies too. The discovery of novel accessible and abundant tumor endothelial-associated antigens, such as those identified in this study, will facilitate the development of more selective anticancer agents based on the targeted delivery of therapeutic molecules to the tumor environment. Furthermore antigens expressed in accessible structures are more likely to be released in circulation upon tissue remodeling and may be detected in the serum [34] or urine of patients with kidney cancer and in turn may be utilized as diagnostics.

## Supporting Information

Table S1 **List of MS identified targets, their tissue source of detection and expression ratio.**
(DOC)
