# Supplementary material for: Identification and Characterization of Angiogenesis Targets through Proteomic Profiling of Endothelial Cells in Human Cancer Tissues
Source: PLoS One. 2013 Nov 13;8(11):e78885. doi: 10.1371/journal.pone.0078885 (PMC3827283; doi:10.1371/journal.pone.0078885)
Supplement: Table S1 — List of MS identified targets, their tissue source of detection and expression ratio. (DOC) [file pone.0078885.s001.doc]

| **Target Symbol** | **Target Name** | **Colon cancer endothelial cells** | **Lung cancer endothelail cells** | **Kidney cancer endothelial cells** | **MIN_RATIO** | **MAX_RATIO** |
| --- | --- | --- | --- | --- | --- | --- |
| LU | Lutheran blood group glycoprotein precursor | - | - | + | 4.7 | 5.8 |
| STEAP4 | STEAP family member 4 | - | - | + | 5 | 10.8 |
| B7-H3 | B7 homolog 3 | - | - | + | 2.5 | 16.2 |
| gi|627559 | pir||A53531 oncofetal trophoblast glycoprotein 5T4 precursor - human | + | - | - | 3.3 | 7 |
| POSTN | periostin, osteoblast specific factor | + | - | - | 4.1 | 4.2 |
| DSG2 | desmoglein 2 | + | - | - | 4.1 | 7.6 |
| CD109 | CD109 antigen (Gov platelet alloantigens) | + | - | - | 3.2 | 3.2 |
| CSPG4 | chondroitin sulfate proteoglycan 4 (melanoma-associated) | + | - | + | 9.7 | 16.1 |
| gi|14602923 | gb|AAH09956.1| HLA-DPA1 protein [Homo sapiens] | - | - | + | 25.8 | 26.7 |
| CD36 | CD36 antigen (collagen type I receptor, thrombospondin receptor) | - | - | + | 2.8 | 16.1 |
| PPAP2B | phosphatidic acid phosphatase type 2B | - | - | + | 6.4 | 6.5 |
| SLC7A2 | Low-affinity cationic amino acid transporter-2 | + | - | - | 7.3 | 7.9 |
| SPARC | SPARC precursor | + | + | + | 5 | 18.6 |
| ITGB1 | Integrin beta-1 precursor | + | - | + | 15.6 | 28.1 |
| MPO | myeloperoxidase | - | - | + | 5.7 | 6.2 |
| LOC51337 | mesenchymal stem cell protein DSCD75 | - | - | + | 4 | 4.7 |
| BST1 | bone marrow stromal cell antigen 1 | - | - | + | 3.4 | 3.7 |
| LAMP1 | lysosomal-associated membrane protein 1 | - | + | + | 2.5 | 17.5 |
| LAMP2 | lysosomal-associated membrane protein 2 | + | - | - | 2.7 | 3.8 |
| IGF2R | insulin-like growth factor 2 receptor | + | - | + | 3 | 8.6 |
| LMAN2 | lectin, mannose-binding 2 | + | - | + | 0.7 | 4.5 |
| XP_114346 | ref|XP_114346.2| similar to Oligosaccharyl transferase 3 [Drosophila melanogaster] [Homo sapiens] | + | - | - | 3.9 | 4.2 |
| ALB | Serum albumin precursor | - | + | - | 53.4 | 69.6 |
| PLAUR | plasminogen activator, urokinase receptor | + | - | - | 9.1 | 11.6 |
| gi|23268459 | gb|AAN11304.1| lactoferrin [Homo sapiens] | - | + | - | 0.195 | 76.9 |
| COPA | coatomer protein complex, subunit alpha | - | - | + | 4.5 | 4.7 |
| gi|230581 | pdb|2IG2|H Chain H, Immunoglobulin G1 | - | - | + | 3.8 | 3.9 |
| HLA-A | HLA class I histocompatibility antigen, A-2 alpha chain precursor | - | - | + | 3.5 | 40.7 |
| AOC3 | amine oxidase, copper containing 3 (vascular adhesion protein 1) | - | - | + | 2.8 | 5 |
| SLC39A8 | solute carrier family 39 (zinc transporter), member 8 | + | - | - | 6.6 | 7.4 |
| LRP1 | low density lipoprotein-related protein 1 (alpha-2-macroglobulin receptor) | + | - | - | 4.5 | 25.2 |
| FBN1 | fibrillin 1 (Marfan syndrome) | + | - | - | 4.8 | 5.8 |
| gi|640301 | pdb|1CDS| Cd59 Complexed With Glcnac-Beta-1,4-(Fuc-Alpha-1,6)- Glcnac-Beta-1 (Nmr, 10 Structures) | - | + | + | 2.2 | 8.9 |
| FBN1 | Fibrillin 1 precursor | + | - | - | 4.8 | 5.8 |
| FN1 | Fibronectin precursor | + | + | - | 0.9 | 62 |
| CPM | Carboxypeptidase M precursor | - | - | + | 3.3 | 21 |
| NID2 | Nidogen-2 precursor | + | - | - | 5.4 | 5.7 |
| MRC2 | mannose receptor, C type 2 | + | - | - | 0.8 | 14.9 |
| gi|21361918 | ref|NP_071751.2| leucine proline-enriched proteoglycan (leprecan) 1 [Homo sapiens] | + | - | - | 4.7 | 10.9 |
| ITGA5 | integrin, alpha 5 (fibronectin receptor, alpha polypeptide) | + | + | + | 0.4 | 23.3 |
| ITGA1 | Integrin alpha-1 | - | - | + | 3.4 | 4 |
| ENPEP | Glutamyl aminopeptidase | - | - | + | 2.9 | 26 |
| CACNA2D1 | Dihydropyridine-sensitive L-type, calcium channel alpha-2/delta subunits precursor | - | - | + | 2.7 | 2.7 |
| HSPG2 | heparan sulfate proteoglycan 2 (perlecan) | + | - | - | 0.6 | 5.7 |
| COL4A2 | Collagen alpha 2 chain precursor | + | - | - | 7.2 | 7.2 |
| LAMA5 | Laminin alpha-5 chain precursor | - | - | + | 2.8 | 18 |
| ENTPD1 | Ectonucleoside triphosphate diphosphohydrolase 1 | - | - | + | 0.9 | 6.3 |
| gi|20452464 | ref|NP_620411.1| endothelial cell adhesion molecule [Homo sapiens] | - | - | + | 5.7 | 5.7 |
| CD200 | OX-2 membrane glycoprotein precursor | - | - | + | 0.3 | 4.7 |
| CDH13 | cadherin 13, H-cadherin (heart) | - | - | + | 3 | 14.9 |
| NMUR1 | neuromedin U receptor 1 | - | - | + | 3.4 | 3.4 |
| INSR | Insulin receptor precursor | - | - | + | 2.7 | 20.6 |
| NT5E | 5'-nucleotidase, ecto (CD73) | - | + | - | 3.681 | 4.113 |
| ITGA6 | Integrin alpha-6 precursor | - | - | + | 2.6 | 4.1 |
| MCAM | melanoma cell adhesion molecule | - | - | + | 6.8 | 14.6 |
| SLC3A2 | solute carrier family 3 (activators of dibasic and neutral amino acid transport), member 2 | + | + | - | 2.9 | 3.7 |
| CD59 | CD59 antigen p18-20 (antigen identified by monoclonal antibodies 16.3A5, EJ16, EJ30, EL32 and G344) | - | + | + | 2.2 | 8.9 |
| PLP2 | proteolipid protein 2 (colonic epithelium-enriched) | + | - | - | 3.1 | 3.9 |
| THY1 | Thy-1 cell surface antigen | + | - | + | 0.1 | 75.5 |
| BSG | basigin (OK blood group) | - | + | + | 2.6 | 117.6 |
| MFI2 | antigen p97 (melanoma associated) identified by monoclonal antibodies 133.2 and 96.5 | + | - | - | 3.5 | 3.7 |
| ADAM9 | a disintegrin and metalloproteinase domain 9 (meltrin gamma) | + | - | - | 3.3 | 10.1 |
| CTL2 | CTL2 gene | - | - | + | 4.2 | 4.3 |
| ITM1 | Oligosaccharyl transferase STT3 subunit homolog | + | - | - | 2.7 | 3.2 |
| GC | Vitamin D-binding protein precursor | - | + | - | 0.944 | 211.289 |
| ICAM1 | intercellular adhesion molecule 1 (CD54), human rhinovirus receptor | + | - | - | 0.2 | 21.5 |
| ITGAV | integrin, alpha V (vitronectin receptor, alpha polypeptide, antigen CD51) | + | - | + | 2.2 | 15.6 |
| ATP2A2 | ATPase, Ca++ transporting, cardiac muscle, slow twitch 2 | - | - | + | 2.7 | 3 |
| CD44 | CD44 antigen precursor | + | + | - | 0.09 | 14 |
| CLIC1 | chloride intracellular channel 1 | - | - | + | 2.5 | 9 |
| THBS1 | Thrombospondin 1 precursor | - | + | - | 14.5 | 22.8 |
| ANPEP | alanyl (membrane) aminopeptidase (aminopeptidase N, aminopeptidase M, microsomal aminopeptidase, CD13, p150) | + | + | + | 0.1 | 8.1 |
| MIF | macrophage migration inhibitory factor (glycosylation-inhibiting factor) | - | + | + | 2.5 | 4 |
| LGALS3BP | lectin, galactoside-binding, soluble, 3 binding protein | + | - | - | 5.3 | 14.9 |
| CYBB | cytochrome b-245, beta polypeptide (chronic granulomatous disease) | - | - | + | 10.8 | 10.8 |
| LTF | Lactotransferrin precursor [Contains: Lactoferroxin A; Lactoferroxin B; Lactoferroxin C] | - | + | - | 0.195 | 76.9 |
| F2 | coagulation factor II (thrombin) | - | + | - | 1.298 | 70.628 |
| ALB | Serum albumin precursor | - | + | - | 53.4 | 69.6 |
| SYPL | synaptophysin-like protein | - | + | - | 3.578 | 4.477 |
| CAPN2 | Calpain 2, large [catalytic] subunit precursor | - | - | + | 2.5 | 2.8 |
| HLA-B | HLA class I histocompatibility antigen, B-44 alpha chain precursor | - | - | + | 6.3 | 12.9 |
| SERPINF2 | Alpha-2-antiplasmin precursor | - | + | - | 7.046 | 8.435 |
| GP25L2 | Glycoprotein 25L2 precursor | - | - | + | 3 | 3.7 |
| CA4 | carbonic anhydrase IV | - | - | + | 3.4 | 3.8 |
| VWF | Von Willebrand factor precursor [Contains: Von Willebrand antigen II] | - | - | + | 2.8 | 5.4 |
| ACE | angiotensin I converting enzyme (peptidyl-dipeptidase A) 1 | - | - | + | 3.8 | 26.6 |
| PECAM1 | Platelet endothelial cell adhesion molecule precursor | - | - | + | 2.5 | 35.9 |
| C3 | complement component 3 | + | + | - | 5.5 | 56.043 |
| PZP | pregnancy-zone protein | - | + | - | 3.885 | 30.7 |
| gi|9716665 | gb|AAF97597.1| nectin 3; PRR3 [Homo sapiens] | + | - | - | 6.4 | 6.8 |
| ITGB1 | integrin, beta 1 (fibronectin receptor, beta polypeptide, antigen CD29 includes MDF2, MSK12) | + | - | + | 15.6 | 28.1 |
| LAMA5 | laminin, alpha 5 | - | - | + | 2.3 | 18 |
| EMP3 | epithelial membrane protein 3 | + | - | + | 2.5 | 4.6 |
| CALU | calumenin | + | - | + | 5.8 | 8 |
| F3 | coagulation factor III (thromboplastin, tissue factor) | + | - | - | 6.7 | 7.3 |
| ITGA3 | Integrin alpha-3 precursor | + | + | + | 2.6 | 4.7 |
| GLG1 | Golgi apparatus protein 1 precursor | + | - | + | 2.9 | 5 |
| ALCAM | activated leukocyte cell adhesion molecule | - | + | - | 2.3 | 5.2 |
| AADACL1 | arylacetamide deacetylase-like 1 | + | - | - | 5.8 | 12.2 |
| ATP1B3 | ATPase, Na+/K+ transporting, beta 3 polypeptide | - | - | + | 4.2 | 5.1 |
| SLC1A5 | solute carrier family 1 (neutral amino acid transporter), member 5 | - | - | + | 2.7 | 2.8 |
| SLC25A3 | solute carrier family 25 (mitochondrial carrier; phosphate carrier), member 3 | + | - | - | 4.3 | 8.4 |
| PLEC1 | plectin 1, intermediate filament binding protein 500kDa | + | - | + | 0.2 | 8.6 |
| BST2 | bone marrow stromal cell antigen 2 | - | - | + | 2.9 | 3.3 |
| AHSG | alpha-2-HS-glycoprotein | - | + | - | 1.861 | 192.839 |
| FER1L3 | fer-1-like 3, myoferlin (C. elegans) | + | - | + | 3.1 | 3.8 |
| CD63 | CD63 antigen (melanoma 1 antigen) | + | - | - | 2.9 | 3.8 |
| ECE1 | endothelin converting enzyme 1 | - | - | + | 3.5 | 3.5 |
| MYADM | Myeloid-associated differentiation marker | + | - | - | 4.9 | 5.2 |
| LOXL2 | lysyl oxidase-like 2 | + | - | - | 7.6 | 8.3 |
| NOMO1 | NODAL modulator 1 | + | - | - | 2.9 | 3.1 |
| FOLH1 | folate hydrolase (prostate-specific membrane antigen) 1 | - | - | + | 1.1 | 22.6 |
| HLA-B | HLA class I histocompatibility antigen, B-45 alpha chain precursor | - | - | + | 6.3 | 12.9 |
| ALB | albumin | - | + | - | 53.4 | 69.6 |
| LNPEP | Leucyl-cystinyl aminopeptidase | - | - | + | 5.6 | 7.6 |
| SSR1 | Translocon-associated protein alpha subunit precursor | + | - | + | 2.9 | 5.6 |
| KIAA0090 | KIAA0090 | + | - | - | 7.6 | 9.1 |
| TIMP1 | tissue inhibitor of metalloproteinase 1 (erythroid potentiating activity, collagenase inhibitor) | + | - | - | 4.3 | 40.8 |
| LOX | lysyl oxidase | + | - | - | 9 | 10.3 |
| ABO | ABO blood group (transferase A, alpha 1-3-N-acetylgalactosaminyltransferase; transferase B, alpha 1-3-galactosyltransferase) | - | - | + | 3.5 | 6.4 |
| ATP1B2 | Sodium/potassium-transporting ATPase beta-2 chain | - | - | + | 3.9 | 4.3 |
| CDH5 | cadherin 5, type 2, VE-cadherin (vascular epithelium) | - | - | + | 2.6 | 5.2 |
| CLEC1A | C-type lectin domain family 1, member A | - | - | + | 3.2 | 3.4 |
| DYSF | dysferlin, limb girdle muscular dystrophy 2B (autosomal recessive) | - | - | + | 2.9 | 5.4 |
| CALCRL | calcitonin receptor-like | - | - | + | 2.8 | 3 |
| LAMA4 | Laminin alpha-4 chain precursor | - | - | + | 22.8 | 24.7 |
| LDLR | low density lipoprotein receptor (familial hypercholesterolemia) | + | - | - | 4 | 4.3 |
